# Vitamin K1-Loaded Poly(Lactic-Co-Glycolic Acid)-Polyethylenimine Nanoparticles Promote Diabetic Wound Healing through FSP1-Dependent Suppression of Ferroptosis

**DOI:** 10.34133/research.1469

**Published:** 2026-10-29

**Authors:** Hongjin Chen, Yiying Chen, Shaowen Cheng, Feiyue Zhao, Kaikai Fan, Sihan Chen, Ying Zhang, Zaijing Liu, Su Wang, Mingzheng Yang, Jiale Song, Xingjie Wu, Jian Wang, Dongliang Yang, Yingnan Song, Xiong Chen

**Affiliations:** ^1^ Center for Tissue Engineering and Stem Cell Research, Translation Medicine Research Center, Guizhou Biomanufacturing Laboratory, Guizhou Medical University, Guiyang, Guizhou, China.; ^2^ Key Laboratory of Emergency and Trauma of Ministry of Education, Key Laboratory of Hainan Trauma and Disaster Rescue, Department of Wound Repair, The First Affiliated Hospital, Hainan Medical University, Haikou, Hainan, China.; ^3^ Department of Pharmacology, School of Basic Medical Sciences, Guizhou Medical University, Guiyang, Guizhou, China.; ^4^ Center of Wound Repair and Regeneration, the First Affiliated Hospital of Wenzhou Medical University, Wenzhou, Zhejiang, China.; ^5^ Department of Wound Repair, the First Affiliated Hospital of Wenzhou Medical University, Wenzhou, Zhejiang, China.; ^6^ Cixi Biomedical Research Institute, Wenzhou Medical University, Ningbo, Zhejiang, China.; ^7^ The State Key Laboratory of Functions and Applications of Medicinal Plants and School of Pharmaceutical Sciences, Guizhou Medical University, Guiyang, Guizhou, China.; ^8^ State Key Laboratory of Flexible Electronics (LoFE) and Institute of Advanced Materials (IAM), School of Physical and Mathematical Sciences, Nanjing Tech University (NanjingTech), Nanjing, Jiangsu, China.

## Abstract

Impaired healing in diabetic wounds presents a major clinical challenge, driven by persistent oxidative stress and dysregulated cellular responses. Herein, a poly(lactic-co-glycolic acid)-polyethylenimine (PLGA-PEI)-based nanoplatform for the sustained delivery of vitamin K1 (VK1) was developed to enhance diabetic wound healing through ferroptosis suppression involving ferroptosis suppressor protein 1 (FSP1). The formulated PLGA-PEI-VK1 nanoparticles exhibited a relatively uniform size distribution, sustained VK1 release, good hemocompatibility, and enhanced antioxidant activity. Network pharmacology analysis suggested that VK1-associated targets were closely related to redox regulation, cell migration, and ferroptosis-associated processes. In vitro, PLGA-PEI-VK1 nanoparticles protected L929 fibroblasts from advanced glycation end products-induced injury; enhanced cell proliferation and migration; attenuated reactive oxygen species accumulation, intracellular Fe^2+^ overload, and lipid peroxidation; restored antioxidant capacity; and increased FSP1 expression. In a diabetic mouse model, PLGA-PEI-VK1 nanoparticles significantly accelerated wound closure and improved tissue regeneration and matrix remodeling, supporting its therapeutic potential in vivo. Mechanistically, molecular docking, cellular thermal shift assay, and molecular dynamics simulations supported an interaction between VK1 and FSP1. Furthermore, pharmacological inhibition of FSP1 markedly impaired the anti-ferroptotic effects of PLGA-PEI-VK1, supporting an important role for FSP1 in VK1-dependent ferroptosis suppression. These findings demonstrate that PLGA-PEI-mediated delivery of VK1 promotes diabetic wound repair, at least in part, through FSP1-associated suppression of ferroptosis and provide a potential nanotherapeutic strategy for diabetic wound management.

## Introduction

Diabetes mellitus (DM) represents a major global public health challenge, with projections from the International Diabetes Federation estimating that approximately 783 million adults will be affected by 2045 [1]. Among the numerous complications associated with DM, diabetic foot ulcers are among the most debilitating, affecting nearly 25% of diabetic patients over their lifetime [2]. These wounds are pathologically characterized by persistent inflammation, impaired angiogenesis, and delayed re-epithelialization. As a result, they progress to chronic non-healing ulcers, and severe cases may ultimately require amputation, thereby imposing substantial physiological and psychological burdens on patients, as well as significant economic strains on healthcare systems [3]. Impaired diabetic wound healing is driven by a multifactorial pathogenesis involving the accumulation of advanced glycation end products (AGEs) and persistent oxidative stress [4]. Although our previous studies showed that accelerating diabetic wound healing is achievable by modulating the wound environment through controlling excessive inflammation and promoting angiogenesis and tissue regeneration, the underlying mechanisms governing cellular metabolic regulation remain unclear [5,6].

In recent years, ferroptosis has emerged as a distinct form of regulated cell death involved in a wide range of pathological conditions, including diabetic complications [7]. This iron-dependent process is driven by excessive lipid peroxidation resulting from disrupted iron homeostasis and impaired antioxidant defense mechanisms [8]. Unlike apoptosis, necrosis, and autophagy, ferroptosis is characterized by specific biochemical and morphological features, including glutathione depletion, inactivation of glutathione peroxidase 4 (GPX4), and extensive lipid peroxidation [9]. In the diabetic wound microenvironment, AGEs induce ferroptotic cell death in endothelial cells, keratinocytes, and fibroblasts, impairing angiogenesis and tissue regeneration [10]. AGEs down-regulate ferroptosis suppressor protein 1 (FSP1) expression in endothelial cells, increasing ferroptosis and reducing angiogenic capacity [11]. FSP1 is a crucial regulator of ferroptosis through its enzymatic reduction of ubiquinone (CoQ10) to ubiquinol, which scavenges lipid peroxyl radicals and suppresses ferroptosis through a GPX4-independent pathway [12]. Accordingly, enhancing FSP1-mediated ferroptosis suppression represents a promising strategy for improving diabetic wound healing.

Vitamin K1 (VK1, phylloquinone) is traditionally recognized for its essential roles in the coagulation cascade and bone metabolism [13]; however, it exerts additional biological activities beyond these classical functions. VK1 possesses antioxidant, anti-inflammatory, and cytoprotective properties across multiple cell types. In addition, VK1 suppresses nuclear factor kappa-B signaling activation, thereby attenuating inflammatory responses in lipopolysaccharide-stimulated THP-1 and RAW264.7 cells [14], alleviates oxidative stress, and enhances cellular viability in retinal pigment epithelium-mediated choroidal neovascularization [15]. VK1 has also been reported to regulate ferroptosis and attenuate oxygen-glucose deprivation-induced neuronal injury [16]. Collectively, these findings suggest that VK1 may hold therapeutic potential in pathological conditions characterized by oxidative stress and dysregulated cell death, including diabetic wounds. However, the clinical application of VK1 remains limited by unfavorable physicochemical properties, including low bioavailability, poor aqueous solubility, and a relatively short plasma half-life. Nanoparticle-based drug delivery systems have been extensively investigated to overcome the pharmacological limitations of conventional formulations [17]. Poly(lactic-co-glycolic acid) (PLGA) has been widely used as an effective drug carrier [18]. Polyethylenimine (PEI), a cationic polymer, enhances cellular uptake and improves nanoparticle stability [19]. The PLGA-PEI hybrid nanoparticle system has demonstrated high delivery efficiency for multiple therapeutic agents, encompassing proteins, nucleic acids, and small molecules [20]. Supporting this, Bivas-Benita et al. [19] reported that PLGA-PEI nanoparticles substantially improve gene delivery efficiency and enhance biological activity in vitro.

In this study, VK1 was loaded into PLGA-PEI nanoparticles to construct PLGA-PEI-VK1, thereby overcoming the delivery limitations of VK1 and improving its therapeutic efficacy in diabetic wound healing. As shown in Fig. 1, AGEs-induced oxidative stress and lipid peroxidation in fibroblasts can promote ferroptotic injury. PLGA-PEI-VK1 suppressed ferroptosis in an FSP1-dependent manner**,** increasing cell survival and promoting fibroblast proliferation and migration, thereby facilitating extracellular matrix remodeling and tissue repair. These results provide experimental evidence supporting VK1-based nanotherapeutic strategies to improve diabetic wound healing.

**Fig. 1.**
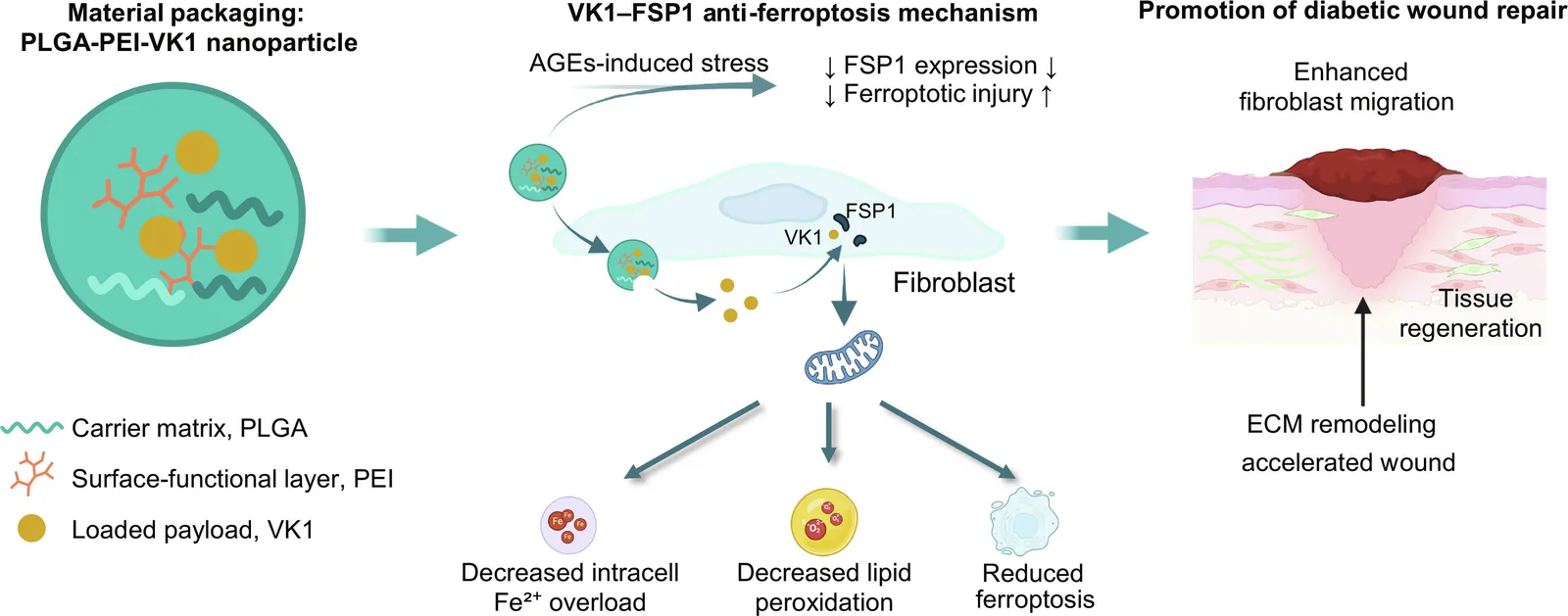
Proposed mechanism of PLGA-PEI-VK1 in diabetic wound repair. In the diabetic wound microenvironment, AGEs-induced stress is associated with reduced FSP1 expression and enhanced ferroptotic injury, characterized by increased ROS accumulation, intracellular Fe^2+^ overload, and lipid peroxidation. PLGA-PEI nanoparticles provide sustained delivery of VK1, which shows target engagement with FSP1, restores FSP1 expression, and enhances FSP1-dependent anti-ferroptotic defense, thereby restoring antioxidant capacity and reducing ROS accumulation, Fe^2+^ overload, lipid peroxidation, and ferroptosis. These protective effects improve the cellular environment for wound repair and are accompanied by enhanced fibroblast migration, extracellular matrix (ECM) remodeling, and tissue regeneration, ultimately accelerating diabetic wound healing. AGEs, advanced glycation end products; ROS, reactive oxygen species; PLGA-PEI, poly(lactic-co-glycolic acid)-polyethylenimine; FSP1, ferroptosis suppressor protein 1; VK1, vitamin K1.

## Results

### Network pharmacology predicts potential ferroptosis-related mechanisms of VK1 in diabetic wounds

The chemical structure and molecular information of VK1 were first retrieved (Fig. 2A), followed by identification of diabetic-wound-related and potential VK1 targets. A total of 9,572 diabetic-wound-related targets and 682 potential VK1 targets were identified, yielding 494 overlapping potential targets (Fig. 2B). A compound–target interaction network was constructed based on the overlapping targets, illustrating the potential interactions between VK1 and diabetic-wound-related proteins (Fig. 2C). Gene Ontology (GO) molecular function enrichment analysis revealed that VK1-associated targets were predominantly enriched in oxidoreductase activity, heme binding, nicotinamide adenine dinucleotide phosphate binding, electron transfer activity, and flavin adenine dinucleotide binding (Fig. 2D). GO biological process analysis further showed enrichment in epidermal growth factor receptor signaling, fibroblast growth factor receptor signaling, cell migration, endothelial cell proliferation and migration, and wound-healing-related processes (Fig. 2E). An integrated target–molecular function association network further demonstrated the close relationships between representative VK1-associated targets and redox-related molecular functions (Fig. 2F). These results indicated a close association between VK1-related targets and redox regulation, a key biological feature associated with ferroptosis.

**Fig. 2.**
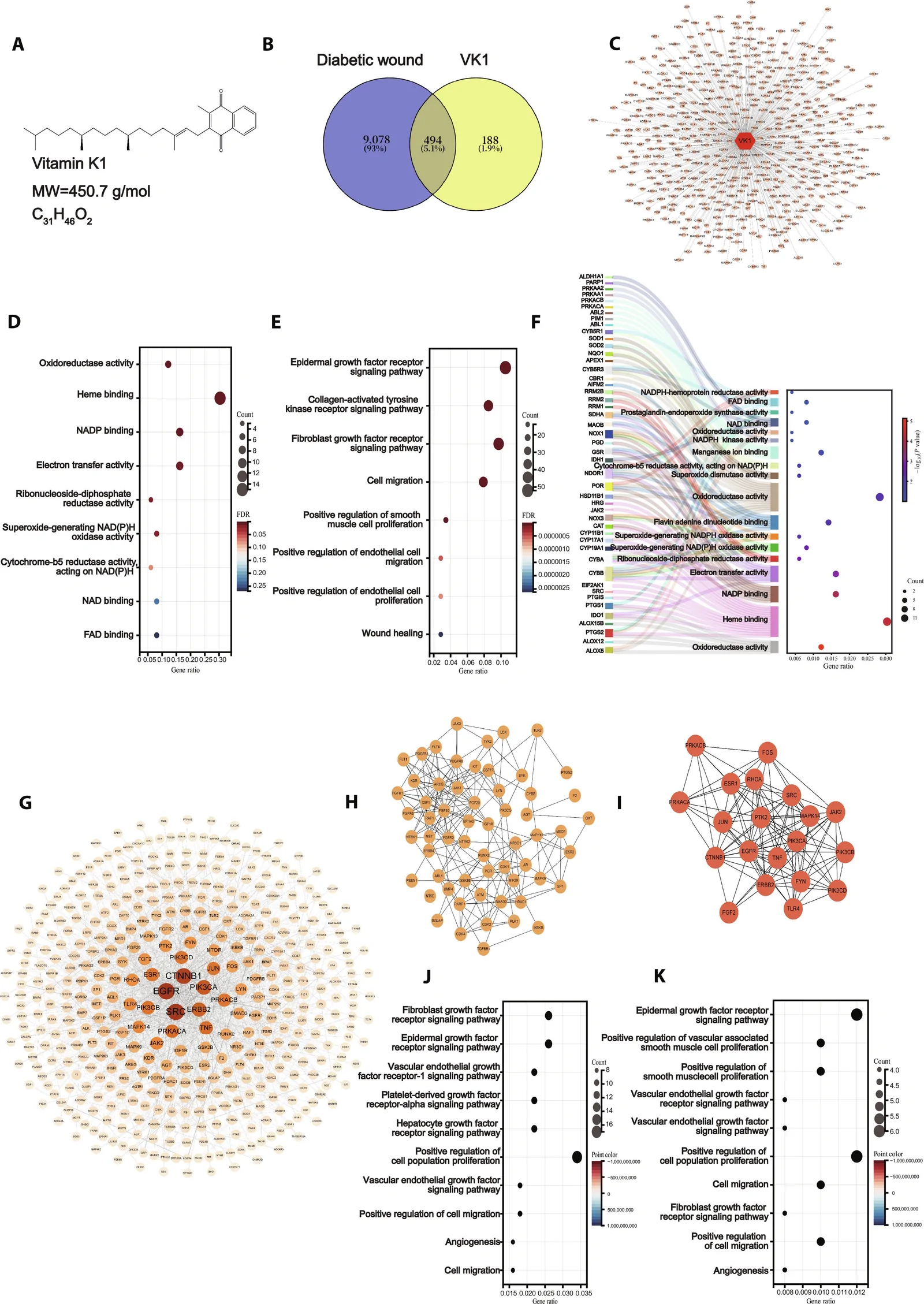
Network pharmacology predicts potential ferroptosis-related mechanisms of VK1 in diabetic wounds. (A) Chemical structure, molecular formula, and molecular weight of VK1. (B) Venn diagram showing the intersection of diabetic-wound-related targets and potential VK1 targets. (C) VK1 compound–target interaction network based on the overlapping targets. (D) Gene Ontology (GO) Molecular Function (MF) enrichment analysis of the overlapping targets. NAD, nicotinamide adenine dinucleotide; NADPH, nicotinamide adenine dinucleotide phosphate; FAD, flavin adenine dinucleotide. (E) GO Biological Process (BP) enrichment analysis of the overlapping targets. (F) Integrated target–molecular function association network illustrating the relationships between representative VK1-associated targets and enriched MF terms. (G) Protein–protein interaction (PPI) network of the overlapping targets. (H and I) Two major functional modules identified from the PPI network by MCODE clustering analysis. (J and K) GO BP enrichment analyses of the 2 MCODE clusters. FDR, false discovery rate.

Further protein–protein interaction network analysis revealed extensive interactions among the overlapping targets (Fig. 2G). Subsequent MCODE clustering analysis identified 2 highly connected functional modules (Fig. 2H and I). GO biological process analysis showed that Cluster 1 was mainly associated with fibroblast growth factor receptor signaling, epidermal growth factor receptor signaling, vascular endothelial growth factor-related signaling, cell proliferation, cell migration, and angiogenesis (Fig. 2J). Cluster 2 was similarly enriched in epidermal growth factor receptor signaling, vascular endothelial growth factor signaling, fibroblast growth factor receptor signaling, cell proliferation, cell migration, and angiogenesis (Fig. 2K). Taken together, these network pharmacology results suggested that VK1 might exert therapeutic effects in diabetic wounds by modulating redox homeostasis and wound-repair-related processes, providing a rationale for subsequent investigation of ferroptosis.

### Characterization of PLGA-PEI-VK1 nanoparticles

PLGA-PEI-VK1 nanoparticles were prepared using an emulsification-based formulation strategy, followed by solvent evaporation, centrifugation, washing, and lyophilization (Fig. 3A). The ability of the nanoformulation to improve the physicochemical properties of VK1 was assessed by characterizing PLGA-PEI-VK1 nanoparticles in terms of particle size and distribution, surface charge, hemocompatibility, and antioxidant capacity. Transmission electron microscopy (TEM) imaging showed that PLGA-PEI-VK1 nanoparticles had a relatively uniform, spherical morphology (Fig. 3B). PLGA-PEI-VK1 nanoparticles had an average diameter of 170.33 ± 12.98 nm (Fig. 3C) and a polydispersity index of 0.220 ± 0.021, indicating a relatively uniform size distribution. In phosphate-buffered saline (PBS) containing 10% fetal bovine serum (FBS), PLGA-PEI-VK1 nanoparticles showed no significant change in size during the first 72 h. The increase in particle size after 72 h may be due to FBS adsorption onto the surface of PLGA-PEI particles and the formation of a protein corona, which can further reduce the particle surface charge and promote nanoparticle aggregation (Fig. S1). In vitro release analysis revealed that approximately 42% of VK1 was released within 24 h, followed by slower release thereafter (Fig. 3D), supporting the suitability of PLGA-PEI-VK1 for sustained delivery.

**Fig. 3.**
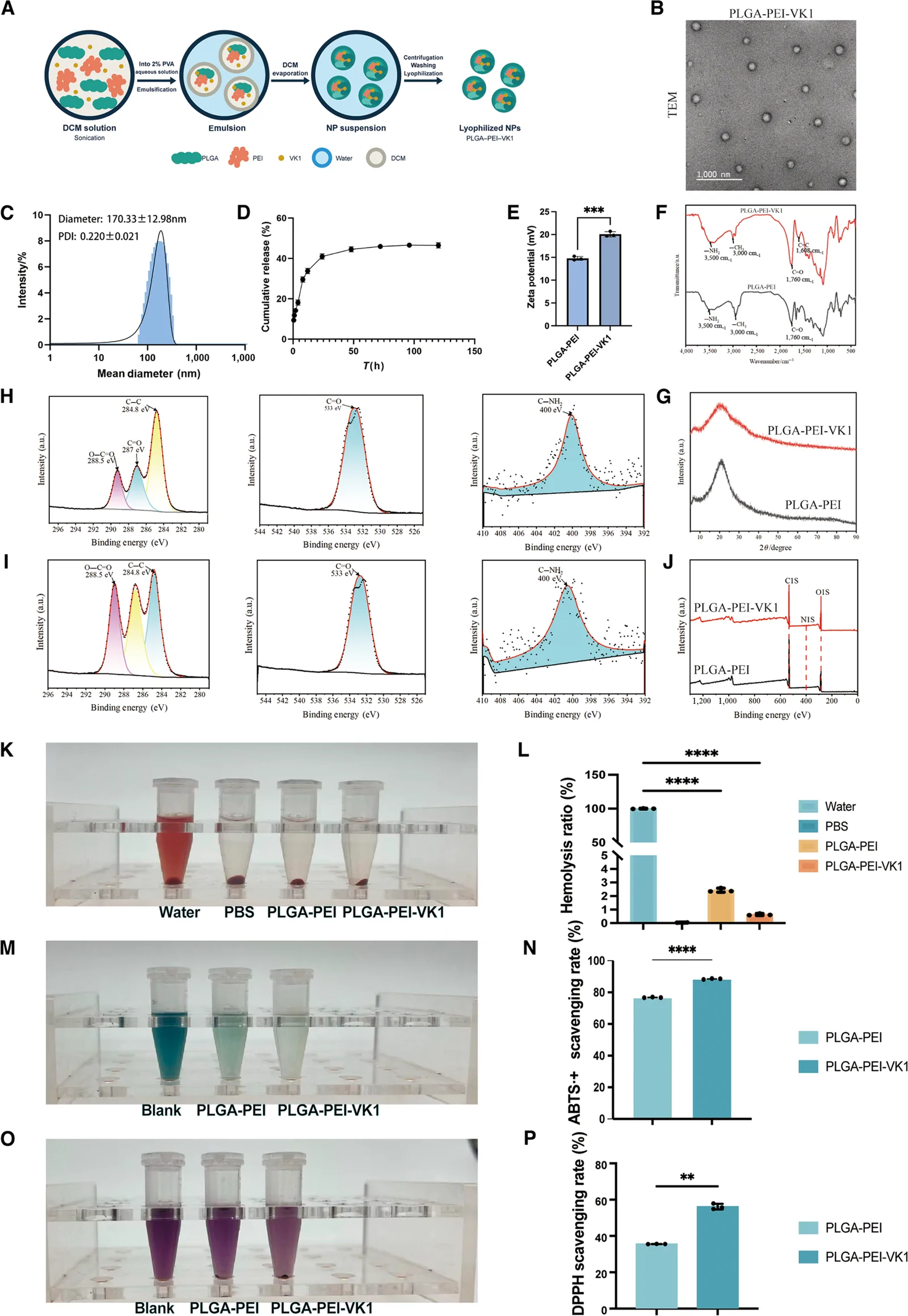
Characterization of PLGA-PEI-VK1 nanoparticles. (A) Schematic illustration of the preparation of PLGA-PEI-VK1 nanoparticles. DCM, dichloromethane; PVA, poly(vinyl alcohol); NP, nanoparticle. (B) Representative TEM image of PLGA-PEI-VK1 NPs. Scale bar, 1,000 nm. (C) Particle size distribution of PLGA-PEI-VK1 NPs. PDI, polydispersity index. (D) In vitro cumulative release profile of VK1 from PLGA-PEI-VK1 NPs. (E) Zeta potential of PLGA-PEI and PLGA-PEI-VK1 NPs. (F) Fourier-transform infrared spectroscopy spectra of PLGA-PEI and PLGA-PEI-VK1 NPs. (G) X-ray diffraction patterns of PLGA-PEI and PLGA-PEI-VK1 NPs. (H and I) High-resolution x-ray photoelectron spectroscopy (XPS) spectra of C 1s, O 1s, and N 1s for PLGA-PEI and PLGA-PEI-VK1 NPs. (J) XPS survey spectra of PLGA-PEI and PLGA-PEI-VK1 NPs. (K and L) Representative images and quantification of the hemolysis ratio. (M and N) Representative images and quantification of the ABTS•+ radical scavenging rate. ABTS, 2,2′-azino-bis(3-ethylbenzothiazoline-6-sulfonic acid). (O and P) Representative images and quantification of the DPPH radical scavenging rate. DPPH, 2,2-diphenyl-1-picrylhydrazyl. Data are presented as the mean ± SD (*n* = 3); **P < 0.01, ***P < 0.001, ****P < 0.0001.

The zeta potential of PLGA-PEI-VK1 nanoparticles was higher than that of blank PLGA-PEI nanoparticles, indicating that VK1 loading altered the surface charge characteristics of the nanoparticles (Fig. 3E). Fourier-transform infrared spectroscopy (FTIR) analysis showed changes in the characteristic spectral features after VK1 loading (Fig. 3F). X-ray diffraction (XRD) analysis demonstrated differences between PLGA-PEI and PLGA-PEI-VK1 nanoparticles (Fig. 3G). X-ray photoelectron spectroscopy (XPS) analysis, including high-resolution C 1s, O 1s, and N 1s spectra, revealed differences in the surface chemical characteristics of PLGA-PEI and PLGA-PEI-VK1 nanoparticles (Fig. 3H and I), while the XPS survey spectra provided additional evidence of differences in surface elemental composition between the 2 formulations (Fig. 3J). Together, these physicochemical analyses support the successful incorporation of VK1 into the PLGA-PEI nanoparticles.

The hemolysis rates of both blank PLGA-PEI and PLGA-PEI-VK1 nanoparticles remained below 3%, indicating good hemocompatibility (Fig. 3K and L). Given the critical role of oxidative stress in diabetic wound pathology, the antioxidant capacity of the nanoformulations was further assessed. Both 2,2′-azino-bis(3-ethylbenzothiazoline-6-sulfonic acid) (ABTS) and 2,2-diphenyl-1-picrylhydrazyl (DPPH) assays showed that PLGA-PEI-VK1 nanoparticles possessed enhanced free-radical scavenging activity compared with blank PLGA-PEI nanoparticles (Fig. 3M to P), confirming the antioxidant activity of the VK1-loaded nanoformulation. These findings suggested that PLGA-PEI-VK1 nanoparticles possessed favorable physicochemical properties, good hemocompatibility, and enhanced antioxidant activity.

### PLGA-PEI-VK1 improves diabetic wound healing in vivo

To evaluate the therapeutic effect of PLGA-PEI-VK1 in vivo, a type 1 diabetic mouse wound model was established, followed by daily subcutaneous administration of PLGA-PEI, PLGA-PEI-VK1, or VK1 formulated in vehicle (dimethyl sulfoxide [DMSO]-VK1), with wound tissues collected at the indicated time points (Fig. 4A). Wound closure was markedly enhanced in the type 1 diabetes mellitus (T1DM)+PLGA-PEI-VK1 group compared with the T1DM+PLGA-PEI group throughout the healing period (Fig. 4B and C). Notably, PLGA-PEI-VK1 also showed a greater wound-healing effect than free DMSO-VK1, supporting the therapeutic advantage of nanoencapsulation.

**Fig. 4.**
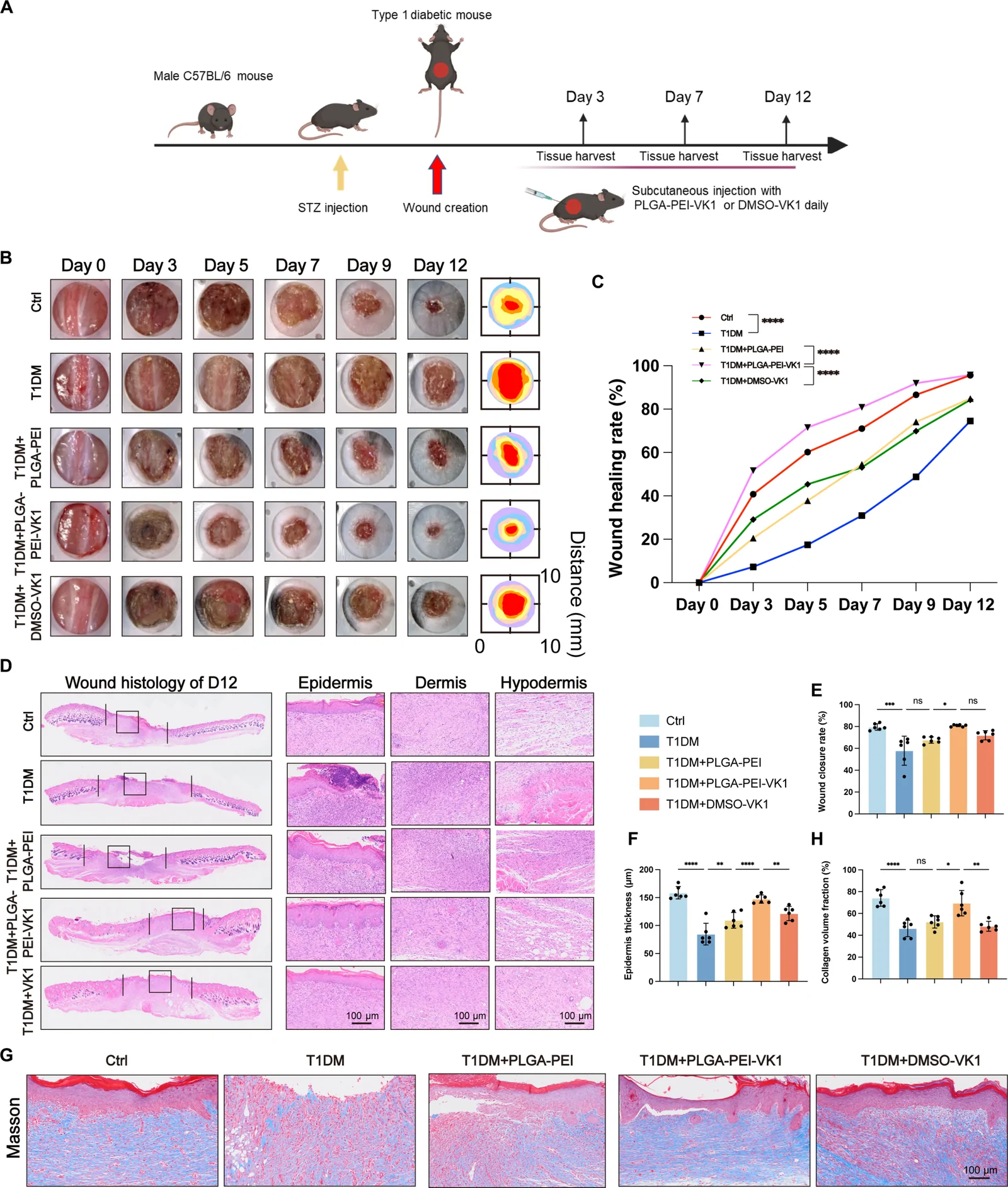
PLGA-PEI-VK1 improves diabetic wound healing in vivo. (A) Experimental protocol and animal grouping. STZ, streptozotocin. (B) Representative images of wound healing at days 0, 3, 5, 7, 9, and 12 in the Ctrl, T1DM, T1DM+PLGA-PEI, T1DM+PLGA-PEI-VK1, and T1DM+DMSO-VK1 groups, with corresponding superimposed wound-area contours illustrating the temporal changes in wound size. (C) Quantitative analysis of wound closure over time. (D) Representative hematoxylin and eosin (H&E)-stained sections of wound tissues collected on day 12, including the epidermis, dermis, and hypodermis. Scale bars, 100 μm. (E) Histological quantification of wound closure on day 12 based on H&E-stained sections. (F) Quantification of epidermal thickness. (G) Representative Masson’s trichrome staining of wound tissues. Scale bars, 100 μm. (H) Quantification of collagen volume fraction. In C, data are shown as mean values (*n* = 6); in E, F, and H, data are presented as the mean ± SD (*n* = 6); *P < 0.05, **P < 0.01, ***P < 0.001, ****P < 0.0001; ns, *P* > 0.05.

Histological evaluation further indicated that PLGA-PEI-VK1 treatment significantly accelerated dorsal skin repair in diabetic mice at day 12 after wounding (Fig. 4D). Quantitative analysis showed that PLGA-PEI-VK1 treatment increased the wound closure rate and restored epidermal thickness compared with the blank PLGA-PEI group (Fig. 4E and F). Masson’s trichrome staining further revealed enhanced collagen deposition in the PLGA-PEI-VK1-treated wounds, accompanied by an increased collagen volume fraction (Fig. 4G and H). The improvement in collagen deposition was also greater than that observed with free VK1, indicating that PLGA-PEI-mediated delivery enhanced tissue remodeling during diabetic wound repair. Taken together, these findings demonstrated that PLGA-PEI-VK1 accelerated wound closure, promoted epidermal regeneration, and enhanced collagen deposition in diabetic wounds.

### PLGA-PEI-VK1 restores AGEs-impaired fibroblast proliferation and migration in vitro

Before comparing the effects of free VK1 and PLGA-PEI-VK1, a concentration–response experiment was performed to establish an appropriate working concentration of free VK1 in AGEs-treated L929 cells. Free VK1 significantly alleviated AGEs-induced cellular impairment within the effective concentration range, with the maximal response observed at 10 μM; this concentration was therefore selected for subsequent free-VK1 experiments (Fig. S2A). These results also confirmed the intrinsic biological activity of free VK1 under AGEs-induced injury.

At the cellular level, AGEs+PLGA-PEI treatment significantly inhibited the proliferation and migration of L929 cells compared to the control group. This impairment was significantly reversed by treatment with PLGA-PEI-VK1, as evidenced by increased cell proliferation in the 5-ethynyl-2′-deoxyuridine (EdU) incorporation assay (Fig. 5A and B). In contrast, blank PLGA-PEI nanoparticles showed no obvious effect on cell proliferation under control conditions, suggesting that the carrier did not impair proliferative activity under these experimental conditions.

**Fig. 5.**
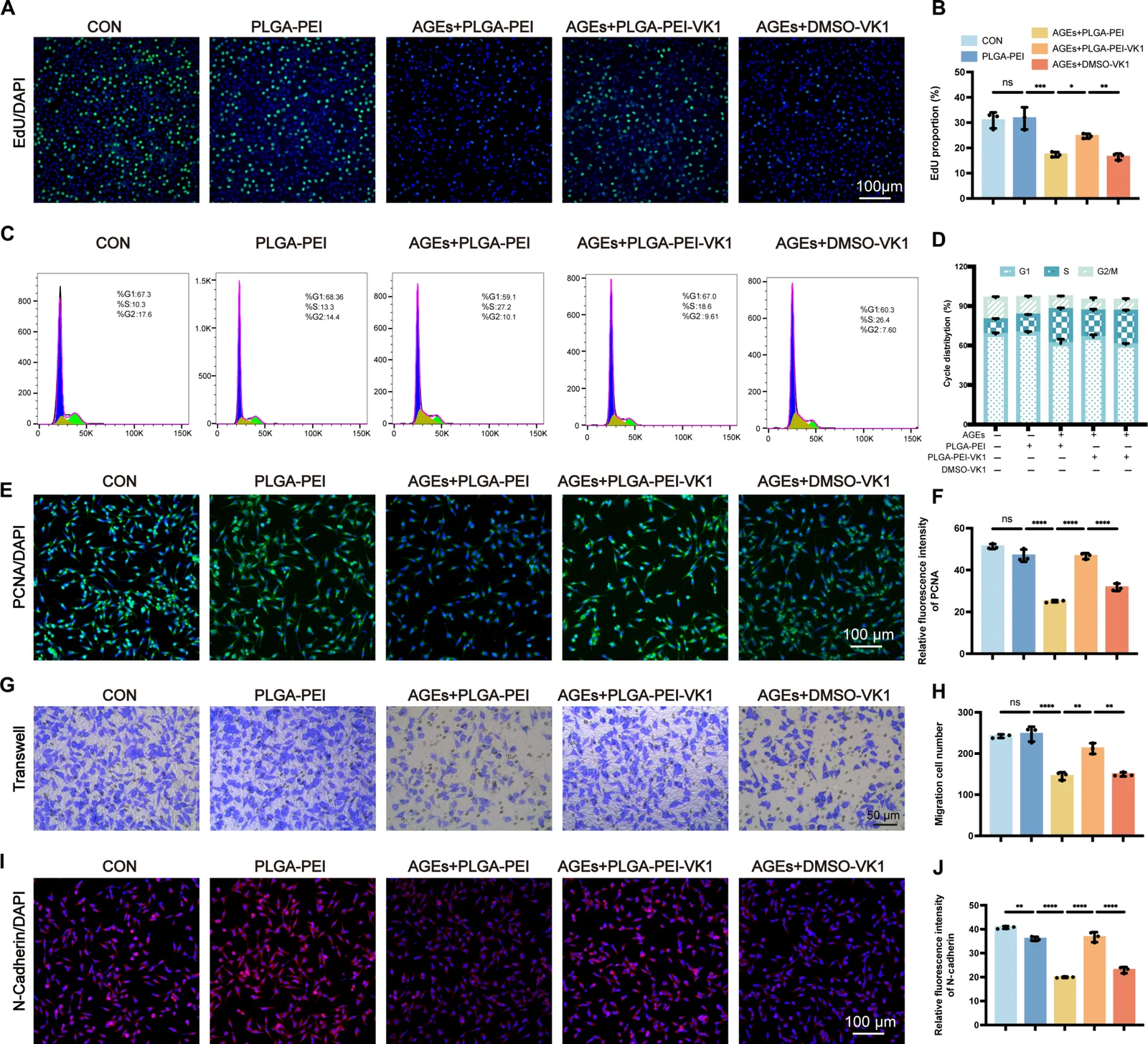
PLGA-PEI-VK1 restores AGEs-impaired fibroblast proliferation and migration in vitro. (A and B) Representative 5-ethynyl-2′-deoxyuridine (EdU)/4′,6-diamidino-2-phenylindole (DAPI) fluorescence images and quantification of EdU-positive cells. Scale bars, 100 μm. (C and D) Representative flow cytometry profiles and quantification of cell-cycle distribution. (E and F) Representative proliferating cell nuclear antigen (PCNA)/DAPI immunofluorescence images and quantification of PCNA fluorescence intensity. Scale bars, 100 μm. (G and H) Representative images and quantification of the Transwell migration assay. Scale bars, 50 μm. (I and J) Representative N-cadherin/DAPI immunofluorescence images and quantification of N-cadherin fluorescence intensity. Scale bars, 100 μm. Data are presented as the mean ± SD (*n* = 3); *P < 0.05, **P < 0.01, ***P < 0.001, ****P < 0.0001; ns, *P* > 0.05.

Flow cytometric cell-cycle analysis further showed that AGEs exposure altered cell-cycle distribution, whereas PLGA-PEI-VK1 treatment partially normalized the cell-cycle profile toward that of control cells (Fig. 5C and D). Consistently, proliferating cell nuclear antigen (PCNA) immunofluorescence staining demonstrated a marked reduction in PCNA expression following AGEs exposure, which was significantly restored by PLGA-PEI-VK1 treatment (Fig. 5E and F), further confirming the recovery of fibroblast proliferative activity. AGEs treatment also markedly reduced the migratory capacity of L929 cells, whereas PLGA-PEI-VK1 significantly increased the number of migrated cells in the Transwell assay (Fig. 5G and H). Additionally, PLGA-PEI-VK1 treatment up-regulated the expression of N-cadherin as determined by immunofluorescence staining (Fig. 5I and J), consistent with the restoration of fibroblast migratory capacity. Notably, although PLGA-PEI-VK1 was administered at a substantially lower VK1-equivalent concentration than free VK1, it produced comparable or greater improvements in EdU incorporation, PCNA expression, cell migration, and N-cadherin expression. These findings suggest that PLGA-PEI-mediated delivery enhanced the apparent cellular efficacy of VK1 under AGEs-induced injury.

### PLGA-PEI-VK1 is associated with mitochondrial respiratory pathway modulation and reduced oxidative stress in diabetic wounds

To further investigate the potential mechanisms underlying the therapeutic effects of PLGA-PEI-VK1, transcriptomic analysis was performed in diabetic wound tissues. Compared with the T1DM+PLGA-PEI group, 270 differentially expressed genes were identified in the T1DM+PLGA-PEI-VK1 group, including 68 up-regulated and 202 down-regulated genes (Fig. 6A). GO biological process analysis showed that these differentially expressed genes were mainly enriched in processes associated with the pyroptotic inflammatory response, inflammasome-mediated signaling, apoptosis, programmed cell death, and regulation of NLRP3 inflammasome complex assembly (Fig. 6B). Molecular function analysis further identified enrichment in GTP binding, GTPase activity, NAD + poly-ADP-ribosyltransferase activity, NAD ± protein mono-ADP-ribosyltransferase activity, calcium ion binding, and pattern recognition receptor activity (Fig. 6C). These findings indicated that PLGA-PEI-VK1 treatment was associated with transcriptional changes involving inflammatory signaling and programmed-cell-death-related processes in diabetic wounds.

**Fig. 6.**
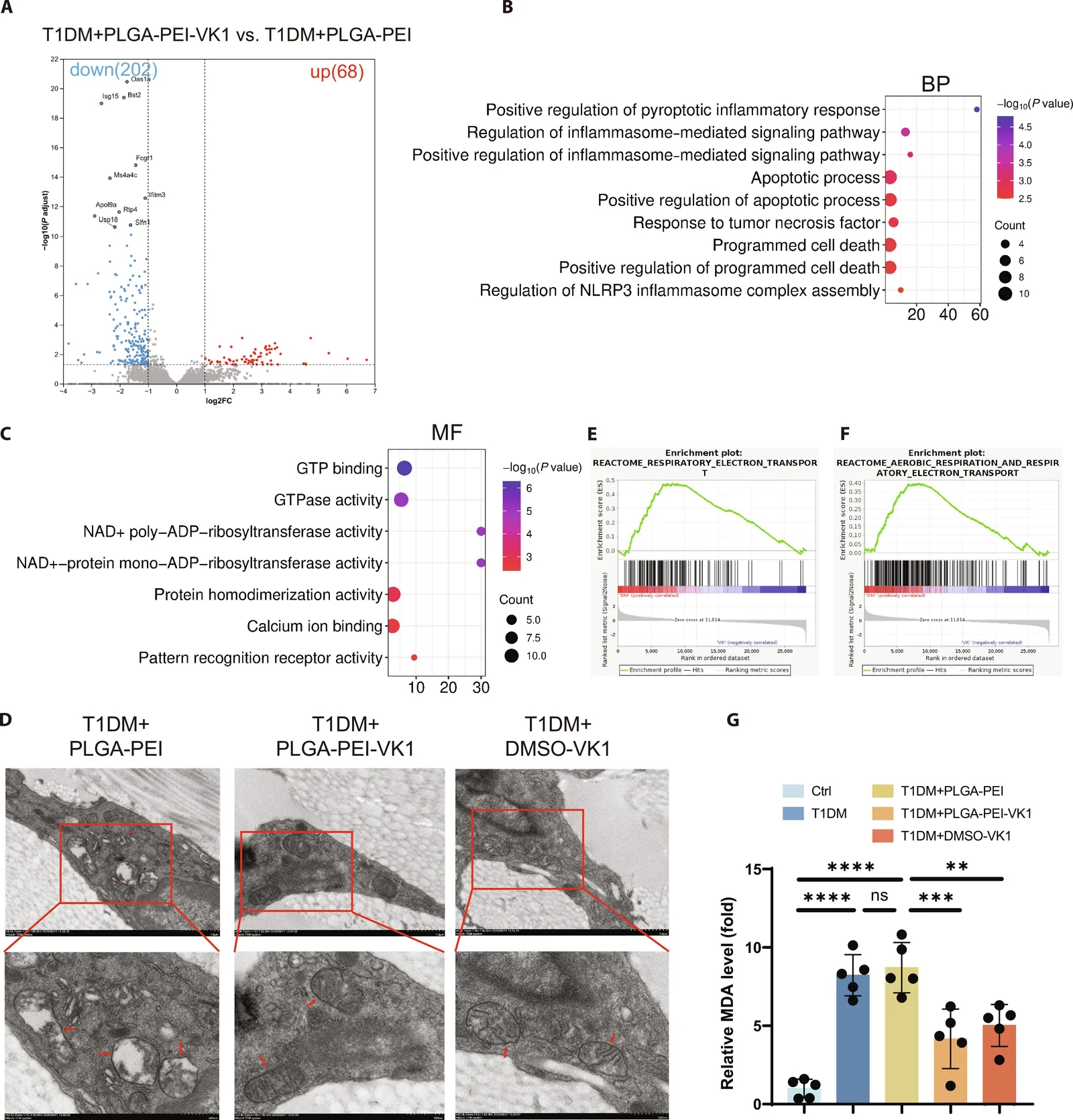
PLGA-PEI-VK1 modulates mitochondrial energy metabolism and oxidative-stress-related processes in diabetic wounds. (A) Volcano plot showing differentially expressed genes between the T1DM+PLGA-PEI-VK1 and T1DM+PLGA-PEI groups, including 68 up-regulated and 202 down-regulated genes. FC, fold change. (B) GO biological process enrichment analysis of differentially expressed genes. (C) GO molecular function enrichment analysis of differentially expressed genes. (D) Representative transmission electron microscopy images showing mitochondrial ultrastructure in the T1DM+PLGA-PEI, T1DM+PLGA-PEI-VK1, and T1DM+DMSO-VK1 groups. Scale bars, 1.0 μm (upper panels) and 500 nm (lower panels). (E) Gene set enrichment analysis (GSEA) of the respiratory electron transport pathway. (F) GSEA of the aerobic respiration and respiratory electron transport pathway. (G) Quantification of malondialdehyde (MDA) levels in wound tissues. Data are presented as the mean ± SD (*n* = 5); **P < 0.01, ***P < 0.001, ****P < 0.0001; ns, *P* > 0.05.

Given the close relationship between mitochondrial dysfunction, oxidative stress, and impaired wound repair, mitochondrial structure and mitochondrial respiration-related transcriptional pathways were further investigated. TEM showed pronounced mitochondrial ultrastructural disruption in the T1DM+PLGA-PEI group, whereas PLGA-PEI-VK1 treatment was associated with improved mitochondrial morphology and better-preserved ultrastructural features (Fig. 6D). Gene set enrichment analysis revealed that respiratory electron transport-related gene sets were preferentially enriched in the diabetic condition relative to PLGA-PEI-VK1 treatment, indicating that PLGA-PEI-VK1 was associated with remodeling of the aberrant mitochondrial respiration-related transcriptional state in diabetic wounds (Fig. 6E and F). These findings suggested that PLGA-PEI-VK1 treatment was associated with modulation of mitochondrial respiration-related pathways.

Consistent with these findings, malondialdehyde (MDA), an indicator of lipid peroxidation and oxidative damage, was markedly elevated in diabetic wound tissues and significantly reduced following PLGA-PEI-VK1 treatment (Fig. 6G). Taken together, these results suggested that PLGA-PEI-VK1 was associated with improved mitochondrial ultrastructure, reduced lipid peroxidation, and modulation of mitochondrial respiratory pathways, which may contribute to the improvement of the diabetic wound microenvironment and facilitate tissue repair.

### PLGA-PEI-VK1 ameliorates AGEs-induced ferroptosis

The effects of PLGA-PEI-VK1 on AGEs-induced ferroptosis were examined to further explore the mechanisms underlying its protective effects in diabetic wound repair. PLGA-PEI-VK1 significantly alleviated AGEs-induced lipid peroxidation, as revealed by reduced MDA levels, while restoring catalase (CAT) activity (Fig. 7A and B). Moreover, AGEs induced reactive oxygen species (ROS) accumulation, which was significantly reduced by PLGA-PEI-VK1 treatment (Fig. 7C, F, I, and J). Consistent with ferroptosis inhibition, PLGA-PEI-VK1 significantly reduced AGEs-induced intracellular Fe^2+^ accumulation and lipid peroxidation (Fig. 7D, E, G, H, K, and L). These findings indicated that PLGA-PEI-VK1 effectively counteracted multiple hallmarks of ferroptosis.

**Fig. 7.**
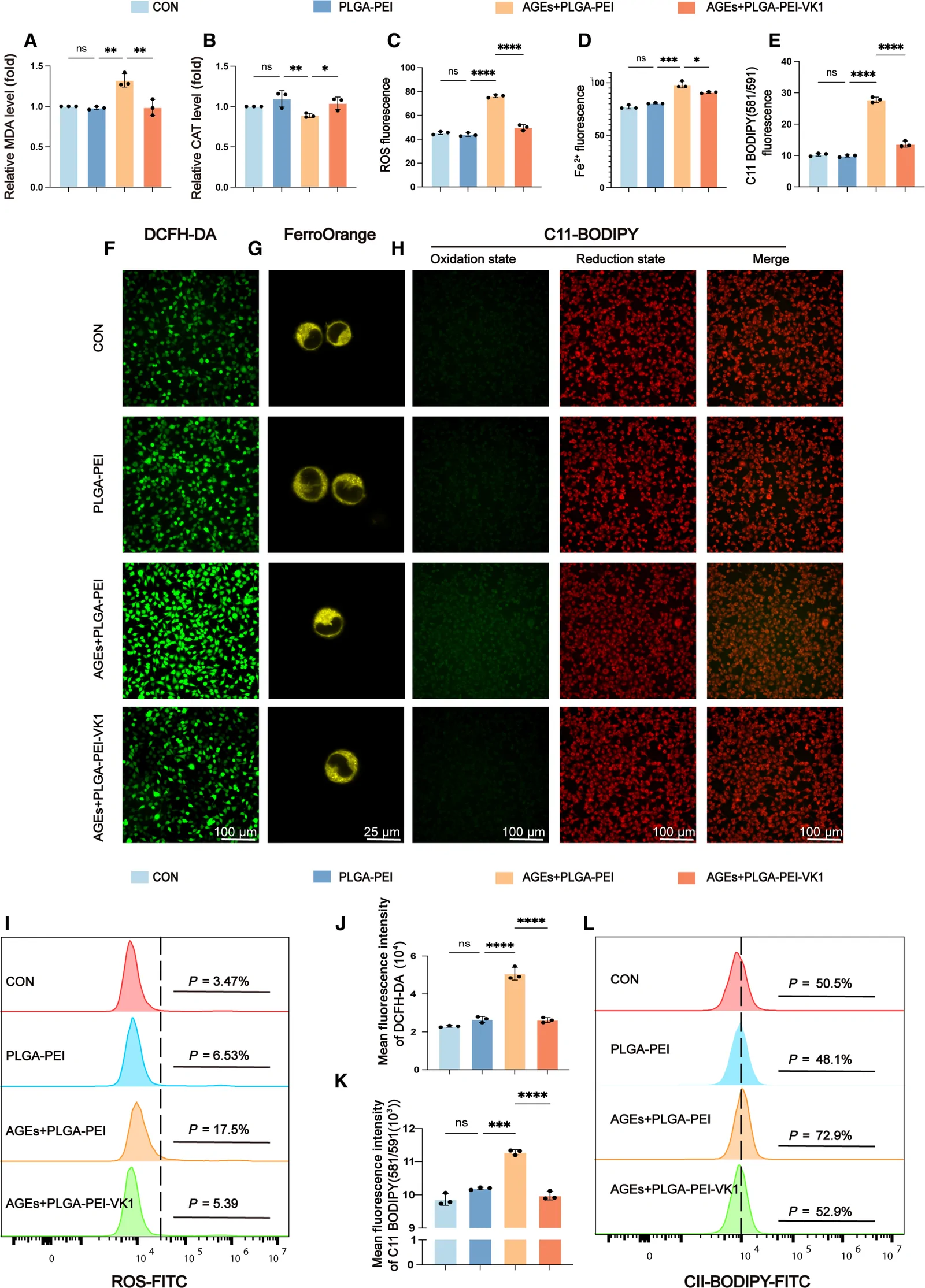
PLGA-PEI-VK1 ameliorates AGEs-induced ferroptosis. (A and B) The relative MDA levels (A) and catalase (CAT) level (B). (C) Quantification of intracellular ROS fluorescence. (D) Quantification of intracellular Fe^2+^ fluorescence. (E) Quantification of C11-BODIPY 581/591 fluorescence reflecting lipid peroxidation. (F) Representative 2′,7′-dichlorodihydrofluorescein diacetate (DCFH-DA) fluorescence images showing intracellular ROS levels. Scale bars, 100 μm. (G) Representative FerroOrange fluorescence images showing intracellular Fe^2+^ levels. Scale bars, 25 μm. (H) Representative C11-BODIPY fluorescence images showing the oxidation state, reduction state, and merged signals. Scale bars, 100 μm. (I and J) Flow cytometric analysis and quantification of intracellular ROS levels. (K and L) Quantification and flow cytometric analysis of C11-BODIPY fluorescence for lipid peroxidation. FITC, fluorescein isothiocyanate. Data are presented as the mean ± SD (*n* = 3); *P < 0.05, **P < 0.01, ***P < 0.001, ****P < 0.0001; ns, *P* > 0.05.

The anti-ferroptosis activity of free VK1 was further evaluated (Fig. S2). Low VK1 concentrations conferred protection against AGEs-induced L929 cell injury, with maximal efficacy observed at 10 μM VK1, which was selected for subsequent studies (Fig. S2A and B). At this concentration, free VK1 reduced MDA levels, restored CAT activity, attenuated intracellular ROS accumulation, and decreased intracellular Fe^2+^ levels in AGEs-treated cells (Fig. S2C to H). AGEs exposure induced pro-ferroptotic signaling characterized by prostaglandin-endoperoxide synthase 2 (PTGS2) up-regulation and concurrent down-regulation of ferritin heavy chain 1 (FTH1) and ferritin light chain (FTL). VK1 administration reversed these molecular alterations by suppressing PTGS2 while restoring the expression of FTH1 and FTL (Fig. S2I to L). These findings collectively demonstrated that VK1 effectively mitigated AGEs-induced ferroptotic cell death by preserving redox balance, iron homeostasis, and ferroptosis-associated regulatory pathways.

### FSP1 is a potential mediator of VK1-dependent ferroptosis regulation

GO cellular component analysis of the candidate targets was performed to characterize their subcellular distribution, and the results showed predominant enrichment in the plasma membrane and other membrane- and cytoplasm-associated compartments (Fig. 8A). Subsequent molecular docking predicted a favorable interaction between VK1 and FSP1, with a binding energy of −8.1 kcal/mol (Fig. 8B). VK1 interacted with FSP1 through contacts involving residues Phe21, Tyr296, Leu300, Thr327, Phe360, and Thr367, with Tyr296 additionally forming a hydrogen bond with VK1. Consistent with the ferroptosis-related changes observed above, AGEs exposure significantly reduced FSP1 protein expression in L929 cells, whereas PLGA-PEI-VK1 treatment restored FSP1 expression (Fig. 8C). Cellular thermal shift assay (CETSA) further showed that VK1 increased the thermal stability of FSP1, consistent with an interaction between VK1 and FSP1 (Fig. 8D).

**Fig. 8.**
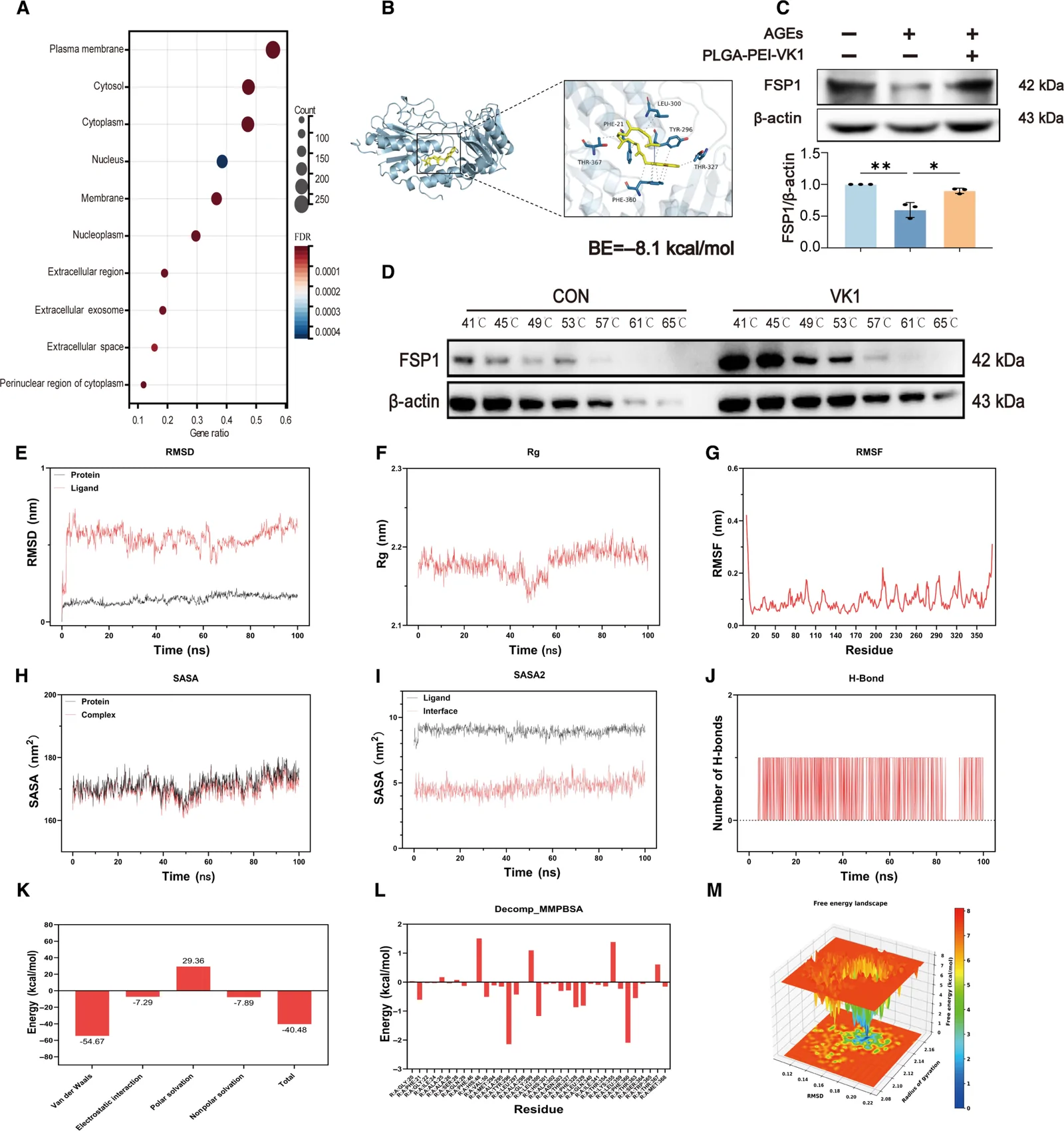
FSP1 is a potential molecular target of VK1 in ferroptosis regulation. (A) GO cellular component enrichment analysis of candidate VK1-related targets. (B) Molecular docking model showing the predicted binding mode of VK1 and FSP1, with a binding energy (BE) of −8.1 kcal/mol. (C) Western blot analysis and quantification of FSP1 protein expression in AGEs-treated L929 cells with or without PLGA-PEI-VK1 treatment. (D) CETSA analysis of FSP1 thermal stability in the absence or presence of VK1. (E) Root-mean-square deviation (RMSD) profiles of FSP1 and VK1 during the 100-ns molecular dynamics simulation. (F) Radius of gyration (Rg) of the FSP1-VK1 complex. (G) Root-mean-square fluctuation (RMSF) distribution of FSP1 residues. (H) Solvent-accessible surface area (SASA) profiles of FSP1 and the FSP1-VK1 complex. (I) Solvent-exposure profiles of VK1 and the FSP1-VK1 binding interface. (J) Number of hydrogen bonds formed between FSP1 and VK1 during the simulation. (K) Molecular mechanics/Poisson–Boltzmann surface area binding free-energy components of the FSP1-VK1 complex. (L) Residue-level binding energy decomposition of FSP1. (M) Free energy landscape of the FSP1-VK1 complex based on RMSD and Rg. For C, quantitative data are presented as the mean ± SD (*n* = 3); *P < 0.05, **P < 0.01.

The dynamic stability of the FSP1-VK1 complex was further supported by molecular dynamics analyses. The root-mean-square deviation (RMSD) of FSP1 remained low and relatively stable after a brief initial equilibration, generally within 0 to 0.2 nm throughout the simulation, indicating overall structural stability of the protein. In contrast, VK1 exhibited greater RMSD fluctuations than FSP1 during the simulation (Fig. 8E). The radius of gyration (Rg) of the FSP1-VK1 complex remained stable within 2.1 to 2.2 nm over the entire 100-ns simulation, indicating a compact and stable overall conformation (Fig. 8F). Root-mean-square fluctuation analysis revealed elevated flexibility at the N-terminal and C-terminal regions, while the central region exhibited relatively low fluctuations, consistent with a stable core structure of FSP1 (Fig. 8G). Solvent-accessible surface area (SASA) analysis showed that the SASA of FSP1 and the FSP1-VK1 complex remained relatively stable throughout the simulation, indicating that VK1 binding did not induce substantial changes in the overall protein conformation (Fig. 8H). The ligand- and interface-associated solvent exposure also remained relatively stable during the simulation, further supporting a stable binding state between VK1 and FSP1 (Fig. 8I). FSP1 and VK1 repeatedly formed a hydrogen bond during the simulation, although the interaction was dynamic rather than continuously maintained (Fig. 8J).

Binding free energy calculations using the molecular mechanics/Poisson–Boltzmann surface area method revealed an estimated binding free energy of −40.48 kcal/mol, indicating energetically favorable complex formation (Fig. 8K). Van der Waals interactions (−54.67 kcal/mol) represented the dominant favorable contribution, with additional favorable contributions from electrostatic interactions (−7.29 kcal/mol) and nonpolar solvation energy (−7.89 kcal/mol) partially offset by unfavorable polar solvation energy (29.36 kcal/mol). Residue-level energy decomposition identified TYR296, LEU300, PHE328, LEU329, and PHE360 as major favorable contributors to VK1 binding (Fig. 8L). Free-energy-landscape analysis based on RMSD and Rg revealed a dominant low-energy basin (RMSD approximately 0.16 to 0.18 nm and Rg approximately 2.12 to 2.14 nm), representing a highly populated and energetically favorable conformational state of the complex (Fig. 8M).

Since GPX4 is the most classical ferroptosis-related protein, its interaction with VK1 was also evaluated. Molecular docking predicted a less favorable interaction between VK1 and GPX4 (−5.1 kcal/mol) than between VK1 and FSP1 (−8.1 kcal/mol) (Fig. S3A). Consistently, neither cell-based western blot nor animal immunofluorescence staining of wound tissue showed restoration of GPX4 levels following VK1 treatment under diabetic conditions (Fig. S3B and C). These findings indicated that GPX4 was unlikely to be the primary mediator of VK1 activity. Together with the restoration of FSP1 expression, CETSA results, and molecular simulation analyses, these findings supported FSP1 as an important molecular mediator and potential target associated with the anti-ferroptotic effects of VK1.

### FSP1 inhibition attenuates the protective effects of PLGA-PEI-VK1 against ferroptosis

Pharmacological inhibition of FSP1 using FSP1 inhibitor (iFSP1) attenuated the protective effects of PLGA-PEI-VK1 against AGEs-induced ferroptosis. In the presence of iFSP1, the ability of PLGA-PEI-VK1 to suppress AGEs-induced increases in MDA, ROS accumulation, intracellular Fe^2+^ accumulation, and lipid peroxidation and to restore CAT activity was markedly attenuated (Fig. 9A to L). Specifically, iFSP1 partially reversed the PLGA-PEI-VK1-mediated reduction in MDA levels and restoration of CAT activity (Fig. 9A and B). Consistently, 2′,7′-dichlorodihydrofluorescein diacetate staining and flow cytometry showed that the reduction in intracellular ROS induced by PLGA-PEI-VK1 was markedly attenuated following FSP1 inhibition (Fig. 9C, D, I, and J). FerroOrange staining further showed that iFSP1 reversed the PLGA-PEI-VK1-mediated reduction in intracellular Fe^2+^ accumulation (Fig. 9E and F). Similarly, boron-dipyrromethene 581/591 C11 (C11-BODIPY) analysis demonstrated increased lipid peroxidation following FSP1 inhibition (Fig. 9G, H, K, and L). These findings indicated that inhibition of FSP1 markedly weakened the anti-ferroptotic effects of PLGA-PEI-VK1.

**Fig. 9.**
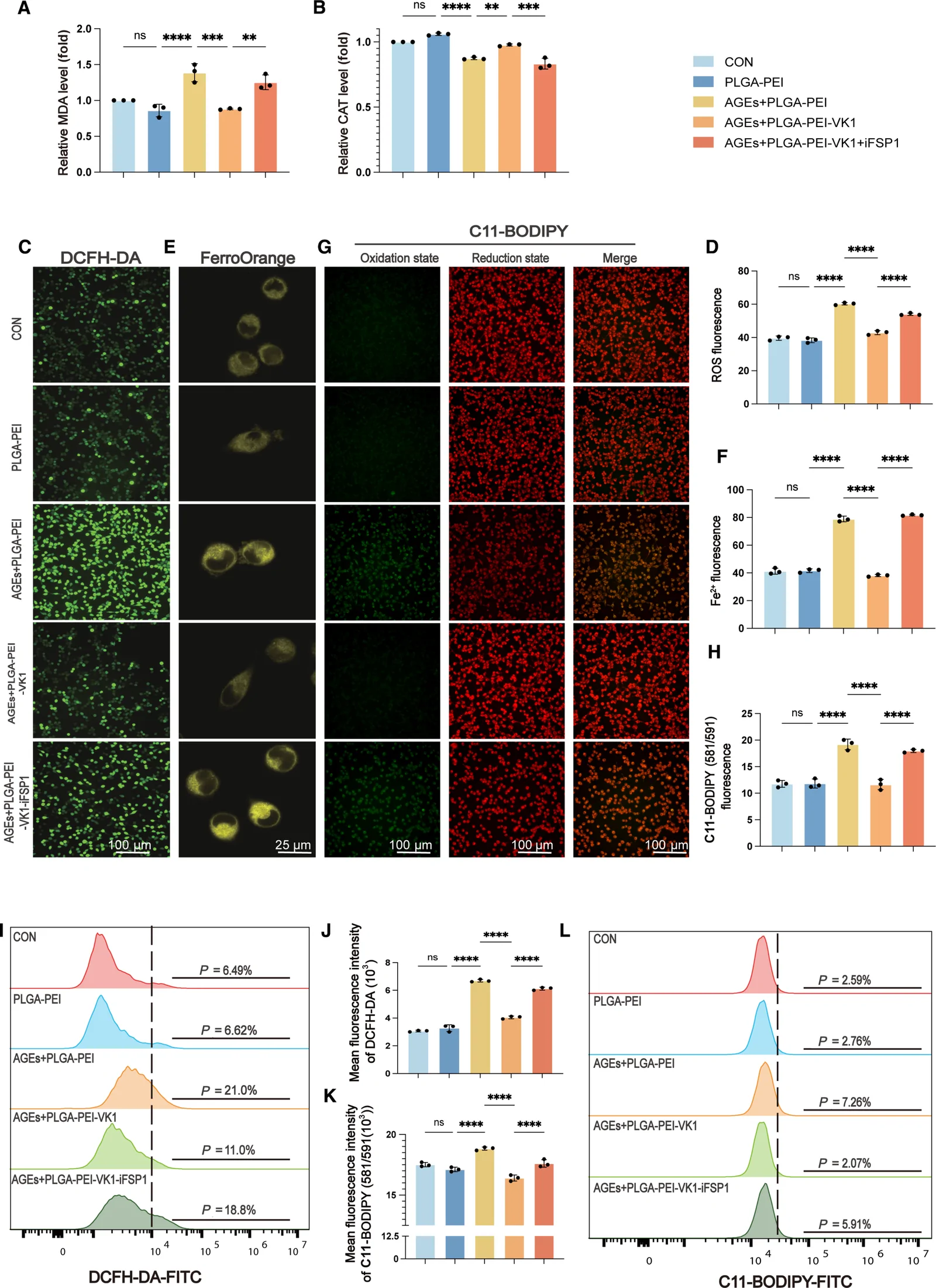
Inhibition of FSP1 attenuates the protective effects of PLGA-PEI-VK1 against ferroptosis. (A and B) The relative MDA levels (A) and CAT level (B). (C and D) Representative DCFH-DA staining and quantification of intracellular ROS levels. Scale bar, 100 μm. (E and F) Representative FerroOrange staining and quantification of intracellular Fe^2+^ levels. Scale bar, 25 μm. (G and H) Representative C11-BODIPY staining and quantification of lipid peroxidation. Scale bar, 100 μm. (I and J) Flow cytometric analysis and quantification of intracellular ROS levels. (K and L) Quantification and flow cytometric analysis of C11-BODIPY fluorescence for lipid peroxidation. Data are presented as the mean ± SD (*n* = 3); **P < 0.01, ***P < 0.001, ****P < 0.0001; ns, *P* > 0.05.

Thus, another FSP1 inhibitor viFSP1 was used to further confirm this effect and produced comparable results by reversing the effects of VK1 on MDA levels (Fig. S4A), CAT activity (Fig. S4B), oxidative stress (Fig. S4C and E), and intracellular Fe^2+^ accumulation (Fig. S4D and F). At the molecular level, viFSP1 partially reversed ferroptosis-associated protein changes induced by VK1 treatment, increasing PTGS2 expression and reducing FTH1 expression, whereas viFSP1 did not significantly alter FSP1 or FTL protein abundance relative to VK1 treatment alone (Fig. S4G to K). These findings collectively demonstrated that pharmacological inhibition of FSP1 substantially impaired the ability of VK1 to suppress AGEs-induced ferroptosis, further supporting FSP1 as an important mediator of VK1-dependent anti-ferroptotic protection.

## Discussion

This study provided evidence that VK1, particularly when delivered through PLGA-PEI nanoparticles, enhanced diabetic wound healing, with the observed therapeutic effects being associated with the suppression of ferroptosis and oxidative stress (Fig. 1). The mechanistic findings further suggested an important involvement of FSP1 in this process. In addition to elucidating a previously underappreciated molecular pathway underlying VK1 activity, the results also demonstrated the sustained-release characteristics of the PLGA-PEI formulation, supporting further investigation of this delivery strategy for diabetic wound management.

Vitamin K status is significantly associated with multiple metabolic and cardiovascular diseases, including chronic kidney disease and coronary heart disease [21,22]. In line with these observations, a concentration-dependent cytoprotective effect of VK1 against AGEs-induced cellular injury was observed, consistent with previous reports describing the antioxidant and cytoprotective properties of vitamin K [15]. The bell-shaped dose–response relationship, characterized by maximal efficacy at intermediate concentrations (5 to 10 μM) and reduced effects at higher concentrations (20 μM), may reflect concentration-dependent differences in VK1-mediated cellular responses rather than a simple linear pharmacological effect. Vitamin K1 attenuates ferroptotic injury through the activation of the Keap1/Nrf2/HO-1 pathway in hepatic models [23]. Another study showed that FSP1 is a warfarin-resistant vitamin K reductase [24] and the vitamin K cycle itself is a potent ferroptosis suppressor [25]. Although the upstream signaling events linking VK1 to ferroptosis regulation remain to be fully elucidated, the present results strongly implicated FSP1 as an important mediator of VK1 activity.

One of the most significant findings in this study was the identification of FSP1 as an important molecular mediator of the anti-ferroptotic effects associated with PLGA-PEI-VK1 treatment. Ferroptosis has recently emerged as a critical contributor to diabetic pathophysiology, including impaired wound healing, where oxidative stress and iron dysregulation are prominent features [8,23]. The FSP1–CoQ10–NAD(P)H axis represents a parallel and GPX4-independent ferroptosis defense system, providing an alternative mechanism by which cells can limit lipid peroxidation and ferroptotic injury [26]. The present results demonstrated that AGEs-induced injury was accompanied by reduced FSP1 expression in L929 fibroblasts, together with increased oxidative stress, lipid peroxidation, and intracellular iron accumulation. PLGA-PEI-VK1 treatment counteracted these changes and increased FSP1 expression. Importantly, pharmacological inhibition of FSP1 attenuated the protective effects of PLGA-PEI-VK1, supporting the involvement of FSP1 in its anti-ferroptotic activity. These findings are consistent with the established role of the FSP1–vitamin K axis in ferroptosis resistance and extend its potential relevance to diabetic wound repair.

The nano-engineering approach used in this study was designed to address formulation-related limitations associated with VK1 delivery. The PLGA-PEI hybrid nanoparticle system combines the biodegradable properties of PLGA with the cationic characteristics contributed by PEI [27]. The prepared nanoparticles exhibited a nanoscale particle size, positive surface charge, and sustained VK1 release in vitro. The positive surface characteristics may facilitate interactions between the nanoparticles and negatively charged cell membranes, although cellular uptake was not directly quantified in the present study. Importantly, the sustained-release behavior observed in vitro suggests that incorporation of VK1 into the PLGA-PEI nanoparticles may prolong its availability compared with rapid release of unformulated drug. Consistent with these formulation advantages, PLGA-PEI-VK1 exhibited greater therapeutic efficacy than DMSO-solubilized VK1 in the present study, supporting the benefit of nanoparticle encapsulation for enhancing the therapeutic performance of VK1. These features are in agreement with recent advances in diabetic wound nanotherapeutics, which highlighted the need for tailored delivery systems capable of addressing the hostile wound microenvironment [28].

From a clinical perspective, the findings of the present work support further investigation of VK1-based therapy for diabetic wound healing. Diabetic foot ulcers represent an enormous healthcare burden, with annual treatment costs exceeding $30,000 per patient in many healthcare systems and persistently high amputation rates despite the current standard of care [2]. VK1 is already clinically used as a hemostatic agent, and its established clinical use may provide an advantage when exploring potential applications beyond its conventional indication. Repurposing a U.S. Food and Drug Administration-approved compound for a new therapeutic indication aligns with current drug development strategies aimed at reducing time, costs, and regulatory barriers [29]. Furthermore, nanoparticle-based delivery may offer advantages in improving the therapeutic performance of hydrophobic agents such as VK1. The modular nature of the PLGA-PEI nanoparticle system also enables future incorporation of additional therapeutic agents, such as antimicrobials or growth factors, thereby supporting a combinatorial approach to address the multifactorial nature of diabetic wound pathology [30].

Several limitations of the present study should be acknowledged. First, although FSP1 was identified as an important mediator, the upstream molecular events through which VK1 regulates FSP1 expression remain undefined. VK1 functions as a ligand for nuclear receptors and may modulate enzymatic activities beyond its classical role in γ-carboxylation [31]. Emerging evidence also suggests that VK1 may influence gene expression through alterations in sphingolipid metabolism and epigenetic regulation [32], representing an important direction for future investigation. Second, while the animal model used in this study is well established, it does not fully capture the chronicity and complexity of human diabetic wounds. Future studies using more complex models, such as diabetic porcine systems with impaired healing, would strengthen translational relevance. Third, long-term nanoparticle stability and effects on wound quality, such as scar formation and mechanical strength, require further investigation. Fourth, although PLGA-PEI-VK1 exhibited greater therapeutic efficacy than DMSO-solubilized VK1 in the present study, the specific mechanisms underlying this formulation-related advantage remain to be fully determined. Further pharmacokinetic and biodistribution studies are needed to clarify whether the improved therapeutic effect results from prolonged VK1 release, enhanced local retention, altered tissue distribution, improved cellular delivery, or a combination of these factors. Finally, although angiogenesis is biologically important for diabetic wound repair and may contribute to the overall healing process, vascular and angiogenic outcomes were not directly investigated in the present study. Therefore, the potential effects of VK1 and PLGA-PEI-VK1 on angiogenesis should be systematically evaluated in future studies.

In conclusion, the present study demonstrates that VK1 exerts therapeutic effects on diabetic wound healing that are associated with FSP1-dependent suppression of ferroptosis and oxidative injury. The PLGA-PEI nanoparticle system enabled sustained VK1 release in vitro and supported the therapeutic effects of VK1 in diabetic wounds. This strategy provides a promising platform for improving the therapeutic application of VK1 in diabetic wound healing, although the pharmacokinetic and tissue-level mechanisms underlying the enhanced efficacy of nanoparticle-encapsulated VK1 warrant further investigation. Beyond wound repair, these findings expand current understanding of vitamin K biology and suggest broader therapeutic implications for diseases characterized by dysregulated ferroptosis.

## Materials and Methods

### Nanoparticle preparation and characterization

PLGA-PEI-VK1 nanoparticles were prepared using a single-emulsion solvent evaporation method. Briefly, PLGA (50:50, molecular weight 30,000 to 60,000) and PEI (molecular weight 25,000) were dissolved in dichloromethane at a weight ratio of 10:1. VK1 was added to the polymer solution at a drug loading of 10% (w/w). The organic phase was subsequently added to a 2% polyvinyl alcohol aqueous solution (w/v) and emulsified by sonication. The resulting emulsion was then subjected to solvent evaporation to remove dichloromethane. Nanoparticles were collected by centrifugation, washed, and lyophilized for further use. Dynamic light scattering (DLS) was used to determine the hydrodynamic particle size, polydispersity index, and zeta potential of the nanoparticles. The morphology of PLGA-PEI and PLGA-PEI-VK1 nanoparticles in the dry state was examined using a Talos L120C transmission electron microscope. Samples were negatively stained with phosphotungstic acid to enhance particle visibility and improve visualization of nanoparticle morphology and boundaries. High-performance liquid chromatography was employed to quantify VK1 loading and encapsulation efficiency. In vitro release studies were performed in PBS (pH 7.4) at 37 °C under shaking at 100 rpm. Particle-size stability in PBS containing 10% FBS was evaluated by DLS at the indicated time points.

### Zeta-potential measurement

The zeta potentials of PLGA-PEI and PLGA-PEI-VK1 nanoparticles were measured using a Litesizer DLS 500 instrument (Anton Paar, Austria). Nanoparticle suspensions were appropriately diluted with deionized water and transferred into zeta-potential measurement cells before analysis. Measurements were performed under identical conditions for PLGA-PEI and PLGA-PEI-VK1 nanoparticles, and the results were expressed as mean ± SD from independent measurements.

### Fourier-transform infrared spectroscopy

The chemical characteristics of PLGA-PEI and PLGA-PEI-VK1 nanoparticles were analyzed by FTIR spectroscopy (SHlMADZU-lRTracer-100, Japan). Lyophilized samples were subjected to FTIR analysis, and spectra were recorded over the appropriate infrared wavenumber range. The characteristic absorption bands of PLGA-PEI and PLGA-PEI-VK1 were compared to evaluate changes associated with VK1 incorporation.

### XRD analysis

The crystalline characteristics of PLGA-PEI and PLGA-PEI-VK1 nanoparticles were evaluated by XRD (Rigaku-Miniflex 600C, Japan). Lyophilized samples were subjected to XRD analysis, and diffraction patterns were recorded over the selected 2θ range. The diffraction profiles of PLGA-PEI and PLGA-PEI-VK1 nanoparticles were compared to assess changes in the physical state of the incorporated VK1.

### XPS analysis

The surface elemental composition and chemical states of PLGA-PEI and PLGA-PEI-VK1 nanoparticles were characterized by XPS (Thermo SCIENTIFIC K-ALPHA, USA). Survey spectra were first acquired to determine the overall elemental composition, followed by high-resolution spectra of C 1s, N 1s, and O 1s. The obtained spectra were analyzed to compare the surface chemical characteristics of PLGA-PEI and PLGA-PEI-VK1 nanoparticles.

### Hemocompatibility assay

Blood samples were collected from C57BL/6 mice into anticoagulant-containing tubes and centrifuged at 3,500 rpm for 15 min to separate red blood cells (RBCs). After removal of the supernatant, the RBCs were resuspended in PBS to obtain a 2% (v/v) suspension. Subsequently, 100 μL of PLGA-PEI-VK1 nanoparticle suspension or blank PLGA-PEI nanoparticle suspension was mixed with 900 μL of the RBC suspension. Ultrapure (Milli-Q) water and PBS were used as the positive and negative controls, respectively. After incubation at 37 °C for 3 h, the samples were centrifuged at 3,500 rpm for 5 min. The absorbance of the supernatant was measured at 540 nm using a microplate reader. The hemolysis rate (HR) was calculated using the following equation:

$$\begin{array}{c} \mathrm{HR}\;\left(\%\right)=\\ \left[\left(\mathrm{OD}\_\text{test}-\mathrm{OD}\_\text{negative}\right)/\left(\mathrm{OD}\_\text{positive}-\mathrm{OD}\_\text{negative}\right)\right]\times 100 \end{array}$$ (1)

### ABTS radical cation scavenging assay

To generate the ABTS radical cation, equal volumes of 7 mM ABTS solution and 2.45 mM potassium persulfate solution were mixed and incubated in the dark at 4 °C for 16 h. The resulting ABTS stock solution was diluted with ultrapure water to obtain an absorbance of 0.70 ± 0.02 at 734 nm. Subsequently, 1 mL of PLGA-PEI-VK1 nanoparticle suspension or blank PLGA-PEI nanoparticle suspension was mixed with 3 mL of ABTS working solution and incubated in the dark for 6 min. The mixture was then centrifuged, and the absorbance of the supernatant was measured at 734 nm (A1). Ultrapure water was used as the blank control (A0). ABTS radical scavenging activity was calculated as follows:

$$\text{ABTS radical scavenging rate}\;\left(\%\right)=\left[\left(\text{A0}-A1\right)/\text{A0}\right]\times 100$$ (2)

### DPPH radical scavenging assay

A 1-mL PLGA-PEI-VK1 nanoparticle suspension or blank PLGA-PEI nanoparticle suspension was mixed with 1 mL of 0.4 mM DPPH solution. The mixture was incubated in the dark for 30 min and then centrifuged. The absorbance of the collected supernatant was measured at 517 nm (A1). Ultrapure water was used as the blank control (A0). DPPH radical scavenging activity was calculated as follows:

$$\text{DPPH radical scavenging rate}\;\left(\%\right)=\left[\left(\text{A0}-A1\right)/\text{A0}\right]\times 100$$ (3)

### Animal grouping and diabetic wound model establishment

Eight-week-old male C57BL/6 mice weighing 18 to 20 g were obtained from Guizhou Medical University. Following a 1-week acclimatization period, the mice were randomly assigned to the following groups: Ctrl mice, diabetic mice (T1DM), T1DM+PLGA-PEI, T1DM+PLGA-PEI-VK1, and T1DM+DMSO-VK1. The number of animals used in each experiment was determined according to the corresponding experimental endpoint and is indicated in the relevant figure or analysis. All animal experiments were conducted in accordance with relevant institutional guidelines and the Animal Research: Reporting of In Vivo Experiments guidelines. Diabetes was induced after a 12- to 16-h fasting period by a single intraperitoneal injection of streptozotocin (100 mg/kg). Blood glucose levels were subsequently measured, and mice with blood glucose levels >11.6 mmol/L were considered diabetic. Following a 1-week stabilization period, full-thickness excisional wounds were created in the diabetic mice. Briefly, dorsal hair was removed using depilatory cream, the skin was disinfected, and a single circular full-thickness wound approximately 1 cm in diameter was generated on the dorsal skin using a biopsy punch. Mice in the corresponding treatment groups received 50 μL of PLGA-PEI or 50 μL of PLGA-PEI-VK1 once daily for 12 consecutive days. For the T1DM+DMSO-VK1 group, an amount of VK1 equivalent to that contained in 50 μL of the PLGA-PEI-VK1 formulation was dissolved in a vehicle consisting of 10% DMSO, 40% PEG300, and 50% normal saline and administered subcutaneously once daily for 12 consecutive days. General health status was monitored daily, and wound healing was photographically documented. The experimental procedure is illustrated in Fig. 4A.

### Wound healing rate calculation

Wound images were captured on days 0, 3, 7, and 12 post-wounding for each experimental group. Wound areas were quantified by ImageJ software, and the wound healing rate was calculated to enable intergroup comparisons with the following formula: Healing rate (%) = [(Initial wound area − Unhealed wound area)/Initial wound area] × 100.

### Hematoxylin-and-eosin staining and Masson staining

Six mice were randomly selected from each group on days 3 and 7 after the start of the treatment, anesthetized with isoflurane, and euthanized. Wound tissue and surrounding skin were harvested. One portion of the tissue was fixed in 10% formalin solution overnight. Following dehydration in a graded ethanol series and clearing in xylene, the tissues were embedded in paraffin blocks for sectioning and staining. Paraffin sections were deparaffinized and rehydrated. Staining was carried out using a modified hematoxylin-and-eosin staining kit or Masson staining kit according to the manufacturers’ instructions. After staining, sections were dehydrated, cleared, and mounted. Morphological changes in the skin and the degree of tissue fibrosis were examined under a light microscope.

### Immunohistochemistry and immunofluorescence staining

Following deparaffinization and rehydration, the paraffin-embedded tissue sections underwent antigen retrieval with a citrate-based buffer under high-temperature and high-pressure conditions. Sections were then blocked with 1% bovine serum albumin (BSA) solution, incubated for 1 h at room temperature, and the exposed primary antibodies diluted in 1% BSA overnight at 4 °C. Details of the primary antibodies are provided in Table S1.

• For immunohistochemistry, sections were incubated with secondary antibody at room temperature for 1 h. The signal was developed using a 3,3′-diaminobenzidine chromogen kit and terminated upon the appearance of brown staining. Finally, sections were counterstained with hematoxylin, mounted, and observed under a microscope.
• For immunofluorescence, sections were equilibrated to room temperature, washed with PBS with Tween 20, and incubated in the dark for 2 h with fluorescent secondary antibodies (Table S1). Following nuclear counterstaining with DAPI for 10 min and washing twice with PBS with Tween 20, sections were mounted in an anti-fade medium for subsequent image with a laser scanning confocal microscope.

### Cell culture and treatment

L929 mouse fibroblasts were purchased from the HonorGene (Changsha, China). Cells were routinely grown in minimum essential medium containing 10% FBS and 1% penicillin–streptomycin. All cultures were kept at 37 °C in a humidified 5% CO_2_ incubator. After stabilization through routine passaging, cells were seeded in 10-cm culture dishes and incubated at 37 °C and 5% CO_2_ until reaching approximately 70% confluence. AGEs at a concentration of 200 μg/mL were used to establish the diabetic stress-related cellular model.

Five treatment groups were established by the random allocation of cells. (a) control (CON); (b) PLGA-PEI; (c) AGEs+PLGA-PEI 5 μL; (d) AGEs+PLGA-PEI-VK1 5 μL; (e) AGEs+PLGA-PEI-VK1 in the presence of 1 μM iFSP1. All groups were incubated for 24 h, and then cells were harvested for mRNA and protein analysis. All experiments were repeated at least 3 times.

### Cell viability

After seeding L929 cells in 96-well plates (5,000 cells/well) and incubating them for 24 h, the cells were incubated for an additional 24 h with various concentrations of VK1 (0, 1.25, 2.5, 5, 10, and 20 μM) and with or without 200 μg/mL AGEs. Cell viability was assessed by employing the Cell Counting Kit-8 (HB-CCK-8-500; Hanbio Tech, Shanghai, China) assay per the manufacturer’s instructions. A microplate reader read absorbance at 450 nm.

### EdU assay

Cells in the logarithmic growth phase were seeded in culture plates. After receiving the corresponding treatments, the medium was replaced with a fresh medium containing 10 μM EdU for 2 to 4 h. The medium was discarded. After a PBS wash, cells were fixed using 4% paraformaldehyde for 15 min and then permeabilized with 0.5% Triton X-100 for 10 min. A freshly prepared click reaction solution (P1226S; Beyotime, Shanghai, China) was added under light-protected conditions and cells were incubated for 30 min to achieve specific EdU labeling. Cells were washed, stained with Hoechst for 10 min, and detected under a fluorescence microscope: nuclei were stained in blue by Hoechst, while EdU-positive cells displayed green fluorescence. Cell proliferation rate was calculated: (EdU-positive cell number/total cell number) × 100.

### Transwell migration assay

A total of 4 × 10^4^ cells in 200 μL were loaded into the upper chambers of Transwell inserts, which had been placed in a 24-well plate containing minimum essential medium with 10% FBS in the lower wells as a chemoattractant. The plate was incubated for 24 h at 37 °C with 5% CO_2_. After incubation, the inserts were removed, and the non-migrated cells on the upper membrane were scraped off with a cotton swab. Cells that had migrated to the lower side of the membrane were fixed by 4% paraformaldehyde and stained with 0.1% crystal violet. The membranes were then washed and examined under a light microscope. Five random fields per membrane were imaged, and the migrated cells in these fields were counted for subsequent quantification and intergroup comparison.

### Measurement of ROS, lipid peroxidation, and oxidative stress

For the measurement of ROS, L929 cells were stained with 2′,7′-dichlorodihydrofluorescein diacetate according to kit instructions (S0043s; Beyotime, Shanghai, China). Briefly, after 3 washes with serum-free medium, cells were incubated with the probe at 37 °C for 30 min and washed again 3 times prior to analysis by fluorescence microscopy and flow cytometry.

Lipid peroxidation was evaluated in vitro using the fluorescent probe C11-BODIPY 581/591. Following a 30-min incubation with 2 μmol/L of the probe at 37 °C in the dark, cells were analyzed via fluorescence microscopy and flow cytometry. The extent of lipid peroxidation was also quantified by measuring MDA levels using a specific assay kit (S0131s; Beyotime). The method is based on the reaction of MDA with thiobarbituric acid at 100 °C to generate a colored/chromogenic product, which was read at 532 nm on a microplate reader. Catalase activity was also measured with a commercial kit (S0051; Beyotime) following the manufacturer’s protocol as an additional indicator of oxidative stress.

### Western blot

L929 cells were first lysed in radioimmunoprecipitation assay buffer supplemented with protease and phosphatase inhibitors. After sonication and centrifugation (12,000 rpm, 10 min, 4 °C), the protein concentration in the supernatant was determined with a BCA kit (20201ES76; YEASON, Shanghai, China). The proteins were diluted in loading buffer, denatured by heating at 100 °C for 8 min, and separated via sodium dodecyl sulfate–polyacrylamide gel electrophoresis. Following electrophoresis, they were transferred onto a polyvinylidene fluoride membrane. The membrane was blocked with 5% skimmed milk for 1 h at room temperature and then probed with primary antibodies (Table S1) overnight at 4 °C. After incubation with the corresponding secondary antibodies (Table S1) for 1 h at room temperature, the signals were detected using an imaging system (BIO-RAD, USA).

### FerroOrange probe assay

L929 cultured cells were washed 3 times using serum-free medium according to the manufacturer’s instructions (F374; Dojindo, Shanghai, China). Then, 200 μL diluted FerroOrange reagent was added, and cells were incubated in a cell culture chamber at 37 °C for 30 min. Images were acquired using a fluorescence microscope.

### Molecular docking

Molecular docking between VK1 and FSP1 was performed using AutoDock Vina. The 3-dimensional structure of VK1 (CAS No. 84-80-0) was obtained from PubChem, whereas the FSP1 protein structure was obtained from the AlphaFold database or Protein Data Bank, as appropriate. Hydrogen atoms were added to the receptor and ligand structures, and the corresponding PDBQT files were prepared using AutoDockTools. Grid-box dimensions and center coordinates were defined according to the receptor structure. Docking simulations were conducted using AutoDock Vina, and the energetically favorable VK1-FSP1 binding conformation was selected for subsequent analysis. Protein–ligand interactions were visualized using PyMOL.

### CETSA–Western blot analysis

L929 cells treated with DMSO or VK1 (10 μM) for 24 h were collected and aliquoted for exposure to different temperatures ranging from 41 to 65 °C for 5 min. Samples were subsequently snap-frozen in liquid nitrogen and processed for protein extraction. Following centrifugation at 12,000 rpm for 30 min at 4 °C, soluble protein fractions were collected and analyzed by Western blotting. FSP1 abundance in the soluble fractions at different temperatures was determined to evaluate the effect of VK1 treatment on FSP1 thermal stability.

### Molecular dynamics simulation

GROMACS 2024.3 software was used to investigate all molecular dynamics simulations. The starting structure was the initial protein–ligand complex from the molecular docking results. Ligand parameters were generated using the Antechamber tool with Austin Model 1-Bond Charge Correction charges and the general AMBER force field force field, while the protein was modeled using the AMBER14SB force field. The Transferable Intermolecular Potential with 3 Points water model was applied to describe the solvent environment. The system was solvated in a cubic simulation box with a boundary distance of 1.2 nm and subsequently subjected to energy minimization using the conjugate gradient method for 50,000 steps. This was followed by constant number of particles, pressure, and temperature equilibration at 310 K and 1 bar for 300 ps and a 100-ns production run. The following analyses were performed on the simulation trajectory: protein and ligand RMSD and root-mean-square fluctuation, Rg, SASA, and hydrogen bond analysis. A free energy landscape was generated based on protein backbone RMSD and Rg. Furthermore, binding free energy was calculated with the molecular mechanics/Poisson–Boltzmann surface area method using 100 frames from the final 50 ns. This calculation employed the Poisson–Boltzmann equation for electrostatic and the SASA model for nonpolar solvation energies. Finally, energy contributions were decomposed for each residue within 6 Å of the ligand.

### Statistical analysis

GraphPad Prism version 8.0 was used for statistical analysis. Data are presented as mean ± SD from independent experiments. For comparisons involving multiple treatment groups at a single time point, one-way analysis of variance followed by Tukey multiple-comparisons test was used. For wound-healing measurements involving both treatment group and time, 2-way analysis of variance followed by Tukey multiple-comparisons test was applied. A *P* value <0.05 was considered statistically significant.

## Ethical Approval

All animal procedures were approved by the Animal Care and Use Committee of Guizhou Medical University, Guian New District, Guizhou, China (No. 2303440).

## Data Availability

The data that support the findings of this study are available from the corresponding authors upon reasonable request.
