# Supplementary material for: Vitamin K1-Loaded Poly(Lactic-Co-Glycolic Acid)-Polyethylenimine Nanoparticles Promote Diabetic Wound Healing through FSP1-Dependent Suppression of Ferroptosis
Source: Research (Wash D C). 2026 Oct 29;9:1469. doi: 10.34133/research.1469 (PMC13716989; doi:10.34133/research.1469)
Supplement: Supplementary file 1 [file research.1469.f1.doc]

**Vitamin K1-Loaded PLGA-PEI Nanoparticles Promote Diabetic Wound Healing through FSP1-Dependent Suppression of Ferroptosis**

HongJin Chen ^1,2,3,4,5 #^, YiYing Chen ^4,5, #^, ShaoWen Cheng ^2, #^, FeiYue Zhao ^6^, KaiKai Fan ^6^, SiHan Chen ^4,5^, Ying Zhang ^1^, ZaiJing Liu ^1^, Su Wang ^1^, MingZheng Yang ^1^, JiaLe Song ^4,5^, XingJie Wu ^7^, Jian Wang ^4,5^, DongLiang Yang ^8,^*, YingNan Song ^1,^*, Xiong Chen ^4,5,6^ *

1. Center for Tissue Engineering and Stem Cell Research, Translation Medicine Research Center, Guizhou Biomanufacturing Laboratory, Guizhou Medical University, Guiyang, Guizhou, China.

2. Key Laboratory of Emergency and Trauma of Ministry of Education, Key Laboratory of Hainan Trauma and Disaster Rescue, Department of Wound Repair, The First Affiliated Hospital, Hainan Medical University, Haikou, Hainan, China.

3. Department of Pharmacology, School of Basic Medical Sciences, Guizhou Medical University, Guiyang, Guizhou, China.

4. Center of Wound Repair and Regeneration, the First Affiliated Hospital of Wenzhou Medical University, Wenzhou, Zhejiang, China.

5. Department of Wound Repair, the First Affiliated Hospital of Wenzhou Medical University, Wenzhou, Zhejiang, China.

6. Cixi Biomedical Research Institute, Wenzhou Medical University, Ningbo, Zhejiang, China.

7. The State Key Laboratory of Functions and Applications of Medicinal Plants & School of Pharmaceutical Sciences, Guizhou Medical University, University Town, Guiyang, Guizhou, China.

8. State Key Laboratory of Flexible Electronics (LoFE) & Institute of Advanced Materials (IAM), School of Physical and Mathematical Sciences, Nanjing Tech University (NanjingTech), Nanjing, Jiangsu, China.

#. These authors contributed equally.

Correspondence: Xiong Chen: xiong_chen@wmu.edu.cn; YingNan Song: yingnansong@gmc.edu.cn; Dongliang Yang: yangdl1023@njtech.edu.cn

**Table S1. Antibody information**

| **Product name** | **Company** | **Catalog** | **Application** | **Dilution** |
| --- | --- | --- | --- | --- |
| FSP1 | Abclonal | A22278 | WB | 1:1000 |
| GPX4 | Abmart | T56959 | WB | 1:1000 |
|  | Abclonal | A1933 | IF | 1:200 |
| PTGS2 | Cell Signaling Technology | 12282 | WB | 1:1000 |
| FTH1 | Abclonal | A19544 | WB | 1:1000 |
| FTL | Abmart | T56955 | WB | 1:1000 |
| N-cadherin | Proteintech | 30803-1-AP | IF | 1:200 |
| PCNA | Proteintech | 10205-2-AP | IF | 1:500 |
| β-Actin | Proteintech | 81115-1-RR | WB | 1:1000 |
| HRP-conjugated Goat anti-Rabbit IgG | Abclonal | AS014 | WB | 1:10000 |
|  |  |  | IHC | 1:200 |
| Goat anti-Mouse IgG (H+L) Secondary Antibody, HRP | Invitrogen | 31430 | WB | 1:10000 |
| Donkey anti-Rabbit IgG (H+L) Highly Cross-Adsorbed Secondary Antibody, Alexa Fluor™ 594 | Invitrogen | A21207 | IF | 1:500 |
| Donkey anti-Rabbit IgG (H+L) Highly Cross-Adsorbed Secondary Antibody, Alexa Fluor™ 488 | Invitrogen | A21206 | IF | 1:500 |

**Fig. S1. Time-dependent stability curve of PLGA-PEI-VK1 nanoparticles.**

**Fig. S2. Effects of free DMSO-VK1 on AGEs-induced cellular injury and oxidative stress in L929 cells.**

(A) Cell viability assay results after treatment with different concentrations of VK1. (B) Cell viability in CON, AGEs, and AGEs+VK1 groups. Data are presented as the mean ± SD (n = 4). Detection of oxidative stress-related indicators: (C-D) The relative MDA levels (C) and CAT level (D). (E, F) Intracellular ROS level detected by DCFH-DA fluorescent probe: (E) Fluorescence microscopy images. Scale bars, 100 μm. (F) Quantitative analysis of ROS level. (G, H) Intracellular iron level detected by FerroOrange fluorescent probe. (G) Fluorescence microscopy images. Scale bars, 100 μm. (H) Quantitative analysis of relative fluorescence intensity. (I) Representative Western blot bands of PTGS2, FTH1, FTL, and β-actin. (J-L) Quantitative analysis of PTGS2/β-actin (J), FTL/β-actin (K) and FTH1/β-actin (L). Data are presented as the mean ± SD (n = 3); *P < 0.05, **P < 0.01, *** P < 0.001, **** P < 0.0001, ns P>0.05.

**Fig. S3. Assessment of the predicted VK1-GPX4 interaction and GPX4 expression.**

(A) Molecular docking model showing the binding interaction between VK1 and GPX4. (B) Western blot analysis of GPX4 expression. (C) Representative GPX4 immunofluorescence images of wound tissues at day 7. Scale bars, 20 μm.

**Fig. S4. viFSP1 Attenuates the Protective Effects of Free VK1 against AGEs-Induced Ferroptosis.**

(A-B) Relative MDA levels (A) and CAT levels (B). (C, E) Quantification (C) and representative DCFH-DA fluorescence images (E) of intracellular ROS levels. Scale bars, 100 μm. (D, F) Quantification (D) and representative FerroOrange fluorescence images (F) of intracellular Fe²⁺ levels. Scale bars, 100 μm. (G) Representative blot bands of PTGS2, FSP1, FTL, FTH1 and β-actin. (H-K) Quantitative analysis of PTGS2/β-actin (H), FSP1/β-actin (I), FTL/β-actin (J) and FTH1/β-actin (K). Data are presented as the mean ± SD (n = 3); *P < 0.05, **P < 0.01, *** P < 0.001, **** P < 0.0001, ns P>0.05.
